# Peanut Allergy and Component-Resolved Diagnostics Possibilities—What Are the Benefits?

**DOI:** 10.3390/nu15245132

**Published:** 2023-12-18

**Authors:** Joanna Połomska, Paulina Dydak, Barbara Sozańska, Hanna Sikorska-Szaflik

**Affiliations:** 1 Department and Clinic of Paediatrics, Allergology and Cardiology, Wroclaw Medical University, ul. Chałubińskiego 2a, 50-368 Wrocław, Poland; joanna.polomska@umw.edu.pl (J.P.); barbara.sozanska@umw.edu.pl (B.S.); 2 Clinical Department of Paediatrics, Specialist Hospital No. 2, Bytom, Silesian Medical University, 40-055 Katowice, Poland; paulina_gora@wp.pl

**Keywords:** peanuts, food allergy, component-resolved diagnostics, anaphylaxis, peanut immunotherapy

## Abstract

Peanut allergy is a widespread and potentially life-threatening condition that affects both children and adults, with a growing incidence worldwide. It is estimated to affect around 1–2% of the population in several developed countries. Component-resolved diagnostics is a modern approach to allergy diagnosis that focuses on identifying specific allergenic proteins to provide precise diagnoses and personalized treatment plans. It is a technique that enables the analysis of specific IgE antibodies against tightly defined molecules (components) that constitute the allergen. Component-resolved diagnostics is particularly valuable in peanut allergy diagnosis, helping to determine allergen components associated with severe reactions. It also aids in predicting the course of the allergy and enables the development of personalized immunotherapy plans; however, the full application of it for these purposes still requires more precise studies. In this paper, we present the current knowledge about peanut allergy and component-resolved diagnostics possibilities. We discuss the possibilities of using molecular diagnostics in the diagnosis of peanut allergy. We focus on examining and predicting the development of peanut allergy, including the risk of anaphylaxis, and describe the latest data related to desensitization to peanuts.

## 1. Introduction

Peanut allergy is one of the most prevalent and potentially life-threatening food allergies worldwide, affecting both children and adults. Over the years, its incidence has been on the rise [1], prompting an urgent need for innovative diagnostic and therapeutic approaches. Among children, the prevalence of peanut allergy has increased, and in European countries, it is estimated at 1.6% [2] and is 2% in the United States [3]. When discussing the epidemiology of peanut allergy, attention should be paid to the methodology of the presented studies, i.e., whether the diagnosis of the allergy was established by a physician based on conducted tests or if it is solely reported by the patient. In the latter case, there may be a significant overestimation of the prevalence of allergy [4,5]. Peanut allergy is usually persistent, continuing into adulthood in ~80% of affected individuals [6]. The rate of patients with peanut allergy experiencing anaphylaxis can vary widely based on several factors, including the severity of the allergy, individual susceptibility, and type of peanut exposure. Peanut anaphylaxis can occur at any age, but it is more commonly seen in children and young adults, likely due to the higher prevalence of peanut allergy in these age groups. According to research, peanuts are responsible for approximately 20% of all anaphylaxes [7,8]. Peanut consumption accounted for 59% of anaphylaxis deaths in the USA and 19% in the UK [9,10].

**Figure 1:**
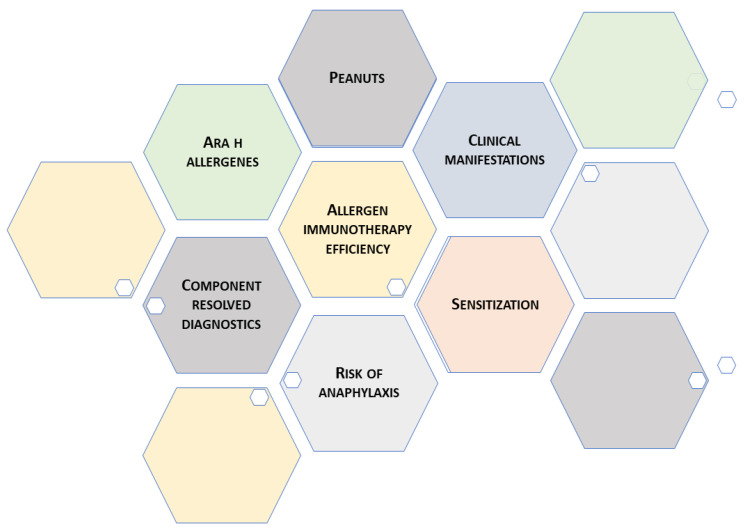
Peanut allergy and component-resolved diagnostics possibilities.

In accordance with the guidelines of the allergology societies, the criterion standard, also called the “gold standard”, for diagnosing food allergy (including peanut allergy) remains an oral food challenge (OFC), ideally a double-blind placebo control food challenge (DBPCFC) [11,12]. The method consists of the exposition of a suspected food-allergic person to the masked version of the appropriately prepared food allergen in increasing dosages and a placebo (on two different days) in a controlled and standardized manner. The challenge concludes when objective symptoms (e.g., vomiting, diarrhea, and urticaria) appear at a specific dose or when the top dose is ingested without evidence of reactivity. The initial dose of 3 mg of food protein (which is 12.5 mg of peanut butter with 24% protein content) seems adequate, and the maximum is usually 3 g of food protein [13]. This method offers advantages, such as minimizing bias by keeping both patients (and, in the case of the pediatric population, their parents) and doctors unaware of allergen administration. This design ensures more reliable, objectively measured results compared to open-label trials. However, drawbacks include resource-intensive requirements, ethical concerns related to potential severe reactions, and limitations in the external validity of findings due to strict control measures. In consideration of the aforementioned factors, such as time consumption and costs, other tests, like the skin prick test (SPT), serum specific IgE (sIgE), basophil activation test (BAT), and serum specific IgE to allergen molecules (CRD), are performed in clinical practice [11]. The basophil activation test is a laboratory technique that utilizes flow cytometry to assess the presence of activation markers (mainly CD63 or CD203c) on the outer surface of basophils in blood [14]. Conducting BAT before an oral food challenge may even allow for the avoidance of the challenge itself. BAT has demonstrated its capability to distinguish patients with clinical allergies from those who are sensitized but tolerant. It exhibits a specificity ranging from 75% to 100% and a sensitivity between 77% and 98% [15,16]. The main advantage of BAT is its low invasiveness compared to provocation tests. In BAT, the activation of basophils in the blood is assessed, eliminating the need to expose the patient to potentially anaphylactic factors, such as peanuts. Another important advantage of BAT is the lack of dependence on antihistaminic drugs, and the limitation may be non-responder basophils excluding up to 40% of samples [17,18]. This method consists of the exposition of a suspected food-allergic person to the masked version of the food allergen in increasing dosages and a placebo (in two different days) in a controlled and standardized manner. Another diagnostic option is measuring specific IgE antibodies for the extraction of a particular allergen. Component-resolved diagnostics (CRD) is a modern approach that enables the analysis of specific IgE antibodies against tightly defined molecules (components) that constitute the allergen. The primary applications of CRD in peanut allergy involve identifying sIgE levels of an individual to specific peanut molecules. This allows for the precise understanding of the main component to which the patient is allergic. The identification of specific components can contribute to assessing a patient’s potential risk of anaphylaxis in response to peanut consumption, as certain components may be associated with a higher likelihood of severe allergic reactions. However, it is important to note that this assessment may not provide absolute certainty. Based on the identification of specific components, a personalized therapeutic plan, including immunotherapy, can be developed. This tailored approach can prove to be more effective and precise, aiming to eliminate or alleviate allergic reactions. With ongoing research, these approaches hold the potential to improve the quality of life of patients with peanut allergy and their families (Figure 1).

## 2. Peanuts-Allergens, Food Allergy Diagnosing and Component-Resolved Diagnostic

Currently, 17 peanut (*Arachis hypogaea*) molecules are known, numbered Ara h 1–Ara h 18, excluding Ara h 4, which was discovered as the Ara h 3 isoform and renamed to Ara h 3.02 [19]. These molecules belong to the following different protein families: cupins (Ara h 1, Ara h 3), conglutins (Ara h 2, Ara h 6, Ara h 7), profilins (Ara h 5), Bet v 1-like (Ara h 8), nonspecific lipid-transfer proteins (nsLTP) (Ara h 9, Ara h 16, Ara h 17), oleosins (Ara h 10, Ara h 11, Ara h 14, Ara h 15), defensins (Ara h 12, Ara h 13), and cyclophilins (Ara h 18). The role of most but not all of them has been described so far [20]. Additionally, not all known peanut molecules can be determined in commonly available tests. Variations in the patient’s profile of sIgE to certain peanut molecules may be demonstrated depending on the region and climate [21].

Allergens belonging to the storage proteins S albumin or S globulin (Ara h 1, Ara h 2, Ara h 3, Ara h 6, Ara h 7) are thermostable, most often cause anaphylactic reactions, and are considered markers of a primary allergy to peanuts (Table 1). 

In the algorithm for the diagnosis of peanut allergy suggested by the British Society for Allergy and Clinical Immunology, serum IgE Ara h 2 + Ara h 8 should be tested in patients with uncertain peanut allergy history with positive SPT/sIgE to whole peanuts [11]. According to American authors of the GRADE analysis and the American Academy of Allergy, Asthma, and Immunology, in a patient with a high probability of peanut allergy, the clinician may use Ara h 2, SPT, or sIgE to confirm the diagnosis, and in patients where there is a low or very low pretest probability of a peanut allergy, no testing is suggested [12]. 

In a meta-analysis of studies on the diagnostic accuracy of peanut component assessment in children with peanut allergy, Nillson et al. found that Ara h 1, Ara h 2, and Ara h 3 are specific to objective peanut allergy. The authors demonstrated that specific IgE to Ara h 2, using the 0.35 kUA/L cutoff, would correctly classify 83.5% of the children with peanut allergy and would give 8.1% false negative results. Simultaneously, specific IgE to Ara h 1 and Ara h 3 would correctly diagnose 62.2% and 64.3% of subjects, respectively [22]. Beyer et al., in a study with 210 children, found that there is a 90% probability that an oral food challenge with peanuts will be positive if the specific IgE to Ara h 2 level is at 14.4 kU/L, and if the level is 42.2 kU/L, then the probability of a positive oral food challenge raises to 95% [23]. In children under 2 years of age, allergy to Ara h 1 is as equally important and common as that to Ara h 2 [24]. Cross-reactivity between Ara h 1, Ara h 2, and Ara h 3 was shown; moreover, co-sensitization to Ara h 2 and Ara h 1 and/or Ara h 3 was found to be predictive of more severe reactions [25].

Other authors suggested that the determination of both Ara h 2 and Ara h 6 has the highest accuracy in detecting peanut allergy [26].

Sensitization to profilin Ara h 5 and Bet v 1-like protein Ara h 8 is related to primary pollen allergy and may be associated with the occurrence of oral allergy syndrome. However, since nuts are consumed mainly after thermal processing, such as being cooked or roasted, the symptoms of this syndrome do not occur so often [20]. Nevertheless, there is a possibility of a severe reaction in patients allergic to Ara h 8 in the absence of antibodies to S albumins/S globulins peanut molecules. Glaumann et al. found that such children can tolerate small amounts of peanuts, but in larger doses, they may be at risk of systemic reactions. The authors suggest that roasting peanuts, as opposed to cooking them, may increase the allergenicity of the Ara h 8 molecule [27].

Ara h 9, Ara h 16, and Ara h 17 belong to the nsLTP family, and they are heat- and gastrointestinal digestive enzyme-resistant. Although adult patients with peanut allergies living in the Mediterranean area are mostly sensitized to Ara h 9, its clinical significance is questionable due to the small content in peanuts (<0.1%) [28,29]. However, according to Brusca et al., in patients that test negative to peanut extract, the measurement of sIgE to Ara h 9 may be a crucial part of peanut allergy diagnosis because of the absence of this molecule in the allergen extract [30].

Ara h 9 shows cross-reactions with other allergens of the nsLTP group, such as peach allergen Pru p 3 or mugwort allergen Art v 3 [24,31]. In clinical practice, patients sensitized to Ara h 9 may only present symptoms related to cross-reactivity to peaches or may remain asymptomatic due to its trace amount in peanuts. The subpopulation of oleosins (Ara h 10, Ara h 11, Ara h 14, Ara h 15) is common energy storage protein in plants. They are also resistant to heat and digestion. Co-sensitization to Ara h 2 occurs, which means that oleosins cannot be treated as the sole allergy trigger, but the presence of anti-oleosin IgE closely correlates to symptom severity [32]. After the roasting process, oleosins are deprived of the protective effect of lipids, which delays peanut digestion and may contribute to a greater risk of severe reactions. This is the reason why oleosins are a candidate biomarker of allergic symptom severity to peanuts [33]. In some countries, sensitivity to oleosins is as prevalent as sensitivity to storage protein; moreover, it is more common among children than among adults [24,33].

Defensins (Ara h 12, Ara h 13) may be associated with severe peanut allergy. As they are small in diameter, they can penetrate the skin barrier. On the other hand, it was proven that defensins have an antifungal effect that could be beneficial for patients with atopic dermatitis using peanut oil-based cosmetics in skin care [34].

Ara h 18 is a peanut cyclophilin. Members of this protein family are highly conserved, and extensive immunological cross-reactivity has been indicated between cyclophilins from different sources. Ara h 18 is about 90% homogenous to Bet v 7, Ole e 15, and Cat r 1. Ara h 18 is rather a cross-reactive molecule, not a primary sensitizer, and it binds IgE antibodies brought out by sensitization to another common allergen source, such as pollen [35].

Although many peanut components are known, only some of them, like Ara h 1, Ara h 2, Ara h 3, and Ara h 6, are used in everyday clinical practice. SIgE to Ara h 2 had the best diagnostic accuracy measures (optimal positive/negative likelihood ratio), and with its very high specificity, it is the best single test for peanut allergy diagnosis because it significantly reduces false positive diagnosis [36]. This is crucial because at least half of preschool children with no history of peanut ingestion and positive SPT to peanut allergens are actually tolerant to peanuts [37]. This leads to the unnecessary avoidance of peanuts, resulting in a lower chance of developing tolerance. According to the authors of the review, Klemans et al., Ara h 2 demonstrated superiority over STP and sIgE to peanut extract. They suggested that this molecular test should replace SPT in clinical practice, especially for children in Northwest Europe, North America, and Australia [36].

Molecular diagnostics enables the identification and quantification of allergenic proteins at a molecular level, allowing for a more precise determination of which peanut proteins the patient is allergic to. Different proteins in peanuts can elicit varying degrees of allergic reactions; thus, understanding the specific allergenic proteins involved is crucial for developing an appropriate treatment plan and managing the allergy effectively. The European Academy of Allergy and Clinical Immunology (EAACI) noted that the CRD is promising but requires further research to find an appropriate place in food allergy diagnosis [12]. 

## 3. Early Peanut Exposure Prevention and Component-Resolved Diagnostic

During pregnancy, the fetus is exposed to food allergens contained in the mother’s diet. Allergens can cross the placenta and be present in amniotic fluid, and after fetal swallowing and suction, they reach the fetal gastrointestinal system and respiratory tract. Regulatory immune responses involving human fetal T cells to both exogenous and endogenous allergens develop as early as 22 weeks’ gestation [38,39]. 

Sicherer et al., in a study with 512 infants enrolled at 3 to 15 months of age, found that the maternal ingestion of peanuts during pregnancy is associated with peanut sensitization, and the sensitization is dose-dependent [40]. In another case-control study, the reported consumption of peanuts during pregnancy and breastfeeding was higher in the group of children with a diagnosed peanut allergy than in controls with no known clinical history or signs of atopic disease [41]. In the Canadian city of Montreal, pregnant and breastfeeding women are advised by many physicians to avoid peanuts. According to a study by Ben-Shoshan et al., in the same city, the prevalence of peanut allergy seems to be stable compared with other developed places where it is still increasing [42].

In the postnatal period, the next source of food allergens is breast milk. The transfer of the peanut allergens Ara h 2 and Ara h 6 into breast milk was reported. There is no conclusion as to whether or not food allergens transferred via breastfeeding to the child are responsible for the development of allergic sensitization or the induction of tolerance [43]. It seems that higher early life exposure to food allergens promotes the induction of tolerance, and the consumption of 2 g/week of peanuts is associated with a significantly lower prevalence of peanut allergy compared with less consumption [38]. In the CHILD cohort, the reduction of peanut allergy by 5 years was noted in infants who were breastfed at the time of early peanut introduction and those whose mother consumed peanuts regularly [44]. According to the EAACI guidelines, there is no evidence that breastfeeding contributes to food allergy prevention [42]. 

Apart from the consumption of peanuts, other factors and compounds that may influence the development of peanut allergy were analyzed. Living with a cat during pregnancy and early life is associated with a threefold increase in the risk of peanut allergy, and cat sensitization is the most common inhalant allergy among 1-year-old children allergic to peanuts. Kotsapas et al. also described the association between early onset and persistent eczema and wheeze and peanut allergy. According to their study, filaggrin loss-of-function mutations in children without eczema is a risk factor of peanut allergy [43]. 

Research conducted thus far suggests that introducing peanuts into the diet early on is beneficial. In a LEAP (Learning Early About Peanut Allergy) study conducted in the United Kingdom, a total of 640 children from age 4 to 11 months who suffered from eczema and/or egg allergy were randomized into two groups that ate or avoided peanuts until the age of 60 months. Peanut consumption was assessed with a questionnaire. Authors of the study found that the early administration of peanuts to children with allergic diseases reduced the risk of developing a peanut allergy [45].

The results of the EAT (Enquiring About Tolerance) study were slightly different. A total of 1303 infants aged 3 months who were exclusively breastfed were randomly assigned to one of two groups, in which six foods with high allergenic potential were introduced into the diet at different times (milk, peanuts, egg, sesame, and white fish meat in any order and wheat last). These products were administered until the age of 36 months. It did not show that the early (from the age of 3 months) introduction of potentially allergenic foods into the infants’ diet reduced the incidence of intolerance to these foods in the first 1–3 years of the child’s life [46].

Ierodiakonou et al. conducted a meta-analysis of the data obtained in the above mentioned studies and concluded that introducing peanuts early reduces the risk of peanut allergy [47].

According to the EAACI recommendations based on many scientific studies, introducing peanuts into the diet should happen between 4 and 6 months of age in children from areas with a high incidence of peanut allergy [42]. However, the American Academy of Allergy, Asthma, and Immunology (AAAAI) recommends that, regardless of the risk of developing allergies, peanuts should be given at about 6 months of age but not before 4 months of age [48]. It is also important that, after introducing peanuts into the diet, they should be eaten fairly regularly [42,48].

Experts advise introducing peanuts into the diet at home. The child must be ready to expand the diet to include these products and should already consume at least two other products. This is important to be able to differentiate possible symptoms, such as coughing and choking, and whether they result from insufficient eating skills or they are a reaction to food, perhaps anaphylaxis [42,48].

Assessing whether a child is allergic to peanuts (by performing SPT or specific IgE testing) before introducing this food into the diet is not applicable. AAAI experts say that some families may insist on such procedures. However, the doctor’s action is then important, and an oral food challenge should be ordered to check whether the patient is actually allergic [48].

The authors of the guidelines did not find an increased risk of peanut allergy in children with older siblings with peanut allergy and do not recommend delaying the introduction of peanuts into the diet. However, they suggest that parents who eliminate peanuts from the diet of an older child may delay the introduction of peanuts to a younger child and thus increase the risk of a peanut allergy in a younger child [48].

It is important to focus not only on peanuts but on diversifying the diet. It was found that a poorly diversified diet in children under 1 year of age was associated with a more frequent diagnosis of food allergies in them [49]. Another idea mentioned in the literature was the introduction of potentially allergenic foods, including peanuts, during breastfeeding [50]. Even though the existing data do not clearly confirm that introducing complementary foods early reduces the risk of allergy, there is no evidence that doing so increases the risk of an allergy. 

Despite all these observations, there is still no laboratory test that can be used to predict the risk of peanut allergy. The development of epitope-specific IgE (ses-IgE) and ses-IgG4 was monitored in children from 4–11 months to 5 years of age as part of the LEAP study. Grinek et al. found that, in children avoiding peanuts, by using the algorithm combining ses-IgE and IgE to Ara h 1, Ara h 2, Ara h 3, and Ara h 9 proteins at 1 year of age, the peanut OFC result at 5 years was predictable [44]. Further research is needed to confirm this hypothesis and formulate evidence-based recommendations. 

The concentration of IgE antibodies against peanuts in adults is typically stable, unlike in children, where it changes over time independently of peanut exposure [51]. This is possibly why the reaction to peanut consumption in children can have a more dynamic nature. To examine the profile of sIgE to certain peanut molecule changes in children, Flinterman et al. measured sIgE for Ara h 1, Ara h 2, Ara h 3, and Ara h 6, conducted DBPCFC, and then repeated the measurements of sIgE after 20 months. Researchers found that, in children with a peanut allergy, Ara h 2 and Ara h 6 were the predominant molecules, and exposure to peanuts during the provocation test did not affect the qualitative or quantitative change in the molecules the child was allergic to. Importantly, only 20 children aged 3 to 15 were included in this study [52]. In the future, it would be worthwhile to assess the concentrations of individual molecules in younger children and their changes over time, especially in a large group of patients. It would also be valuable to evaluate how an elimination diet or the deliberate exposure to peanut allergens affects the concentration of sIgE against specific molecules. Conducting a study illustrating whether and how the patient’s profile of sIgE to certain peanut molecules changes would allow for a better understanding of peanut allergy.

## 4. Peanut-Induced Anaphylaxis and Component-Resolved Diagnostic

Traditionally, anaphylaxis is known as a severe, multisystemic, and potentially life threatening hypersensitivity reaction regardless of the trigger or underlying mechanism (immunological, involving IgE, IgG, or immune complexes, or non-immunological) [53]. The results of many recent surveys conducted in developed countries revealed the increasing prevalence of anaphylaxis, but it is still unknown whether this is related to increased exposure to a given allergen or changes in dietary habits (vegan/plant-based diet) [54].

According to the European Anaphylaxis Registry, based on the research conducted in ten European countries between July 2007 and March 2015, food allergies were responsible for 66% of the reported cases of anaphylaxis in the pediatric population, with peanuts as a prevalent elicitor at all ages [55]. It is challenging to estimate the frequency of anaphylaxis to peanuts due to diverse diagnostic criteria and overlapping nomenclature used among different countries. The most common diagnostic criteria were recently proposed by the EAACI guidelines in 2021, including the concept of likely and/or known allergen in its criteria and the World Allergy Organization (WAO) guidelines 2021 [56,57].

Reactions to peanuts typically manifest initially in childhood, with symptoms usually within the first 2 years of life and with the cutaneous system affected in 89% of individuals and the respiratory system affected in 42% of individuals during the first presentation, often occurring on the first known exposure, which is most common at home [58,59]. Some studies indicate that only twenty percent of children will naturally outgrow their allergy to peanuts, which means that it tends to persist throughout life, and most children allergic to peanuts will not tolerate them later on in life; the severity of reactions to peanuts may even increase with age. Asthma and delay in administering epinephrine in anaphylaxis are risk factors for a poor outcome of peanut anaphylaxis [60,61]. It represents one of the most common causes of food-induced hospital admissions and deaths. The analysis of national 1998–2018 data relating to hospital admissions for anaphylaxis and deaths, and prescription data for adrenaline autoinjector devices in the United Kingdom population, demonstrated that at least 46% of deaths were triggered by peanuts or tree nuts [62,63].

The diagnosis of a peanut allergy consists of the medical history of a peanut-induced allergy reaction (type/quantity of food ingested, the time of symptom onset, severity and duration of symptoms, medical treatment, personal/family atopy), physical examination, and diagnostic tests. SPT with commercially prepared food extracts has a number of limitations in the diagnosis of a peanut allergy. It was proven that infants and very old individuals are less likely to develop adequate control wheals [64]. What is more, SPT and peanut-specific IgE levels do not predict clinical severity.

Peanuts, or *Arachis hypogaea* L., that originate in South America botanically are not true nuts but “ground nuts” and belong to the legume family, along with peas, beans, chickpeas, soybeans, alfalfa, clover, lentils, beans, lupine, and fenugreek. Scientific reports revealed that, in France, approximately 15% of food-related anaphylaxis in the pediatric population is caused by legumes, and among them, the most common trigger is peanuts, which are responsible for almost 80% of cases. The determination of the prevalence and relevance of sensitization to legumes in peanut-allergic children is needed because of the increasing consumption of legumes, data on cross-reactivity between peanuts and other legumes, and frequent severe allergic reactions observed in children with a peanut allergy [65,66].

Patients with a peanut allergy may have positive results of SPT or serum tests to tree nuts such as hazelnut or chestnut that are known as true nuts because they contain an edible seed with a hard shell and woody protective layer. This may be explained by IgE cross-reactivity, defined as the relationship between at least three reagents: cross-reactive antibody and two allergens, based on the similarity of their epitopes (cross-reactive epitopes). The closer the similarity between two proteins, the more likely allergen cross-reactivity occurs, but it is only possible to estimate the probability of cross-reactivity, and what is more, the cross-reactivity may be clinically important or irrelevant [67].

Relevant cross-reactivity between tree nuts and peanuts has been well demonstrated in sensitized patients, but not all peanut-allergic individuals present severe responses to oral exposition. The results of a recent study on peanut-sensitized patients showed that most of them avoid tree nuts, whereas CRD demonstrated that only part of them presented species-specific sensitizations to tree nuts; therefore, most peanut-allergic individuals could potentially reintroduce tree nuts into their diet. The limitation of the study was that the oral food challenge with tree nuts was not performed to determine the clinical significance of these findings [68]. The total protein content of peanuts consists of allergens such as seed storage proteins of the 2S albumin, the vicilin protein family, and the legumin protein family, and homologue allergens with IgE-cross-reactive epitopes belonging to these protein families were also identified for other legumes and tree nuts, which may be an explanation for the observed co-sensitization [69,70,71].

Knowledge of the cross-reactivity between similar epitopes of homologue proteins should be implemented in the area of allergen-specific immunotherapy to induce tolerance to different food sources of allergens simultaneously [72].

In peanuts, Ara h proteins provoke a strictly IgE-mediated type I hypersensitivity reaction, causing immediate symptoms that can range from mild reactions to severe anaphylaxis that have tendency to be severe, although the severity may vary with different episodes of exposure [58,73]. CRD, based on recombinant protein immunoassays, can elucidate which of the Ara h proteins the patient’s antibodies are reactive to and, therefore, can predict the clinical severity of the patient’s allergy [68]. According to current knowledge, allergies to certain molecules carry a higher risk of developing a severe allergic reaction. Individuals with sIgE to the seed storage proteins Ara h 1 (vicilin), Ara h 2 (2S albumin), and Ara h 3 (legumin) are at higher risk of severe systemic reaction after peanut exposure than individuals with sIgE to Ara h 8 (pathogenesis-related (PR) protein homologous to Bet v 1 and other birch pollen allergens), who are more likely to develop milder oral allergy syndrome [74].

A clinically significant reaction, including anaphylaxis after exposure to peanuts, is possible in patients who do not have positive SPT results (diagnostic gap caused by allergy to glycosyltransferase (Ara h 10/11, 14/15) and defensins (Ara h 12/13)). Glycosyl transferase, also called oleosin, are hydrophobic proteins that are not present in aqueous allergen extracts used for diagnostics. An isolated allergy to oleosins is possible, and in some populations, an allergy to peanut oleosins is as common as an allergy to storage protein allergens [20,33].

CRD seems to be a valuable complement to other diagnostic tools, such as medical history focused on symptoms after peanuts consumption and SPT/sIgE with peanut extract. Efforts should be undertaken to develop molecular allergy diagnostic methods to help estimate the risk of allergic reaction in people allergic to peanuts and avoid unnecessary elimination diets in people with allergies of low clinical significance.

## 5. Peanut Immunotherapy and Component-Resolved Diagnostics

Tremendous progress in food allergy management has been made because of the increasing understanding of how desensitization occurs; nowadays, allergen exposure may be used not only as a prevention of food allergy during pregnancy and early childhood but also in desensitization to food allergens [75]. In the past, peanut avoidance or the treatment of peanut allergen-induced systemic reactions with adrenaline remained the standards of care for sensitized individuals. However, so many food products are labeled as containing peanuts; therefore, it is not easy for patients allergic to peanuts to follow the recommended elimination diet, and the constant need for surveillance significantly limits their everyday life [76].

Allergen immunotherapy is a form of therapeutic management that involves the repeated administration of allergen extracts or recombinant allergens for established IgE-mediated hypersensitivity to modify the immune response upon the allergens and provide long term relief of symptoms. Among the underlying mechanisms, there are the induction of regulatory T and B cells, the production of antibodies IgG and IgA, as well as the reduction of allergen-specific T helper 2 cells [77]. Nowadays, allergen-specific immunotherapy for peanut allergy has been better researched than immunotherapy for other food allergies and has been pushed to the forefront as a treatment option. Modern immunotherapy for peanuts may be categorized as oral immunotherapy (OIT), sublingual immunotherapy (SLIT), or via the skin, as epicutaneous immunotherapy (EIT) [78].

Worth noticing is that a study on subcutaneous peanut immunotherapy was conducted in 1992 to establish the efficacy and safety of rush immunotherapy with peanut extract. The reduction in symptom scores during the double-blind, placebo-controlled, peanut challenge and the decrease in end point-titrated PSTs to peanuts were observed [79]. Another study on the effect of injections of peanut extract in achieving desensitization to peanuts was associated with a higher rate of repeated systemic reactions, which demonstrated that this method was not acceptable for routine use [80]. It would be interesting if, now, several decades later, by using component diagnostics, we could characterize the sensitization pattern of individual study participants and, on this basis, assess their individual risk of a severe reaction to injected peanut extract.

The aim of immunotherapy for peanuts is to induce tolerance to a dose of peanuts several times higher than the dose that previously caused an allergic response and, therefore, to reduce the risk of a life-threatening allergic reaction. The main benefit of peanut immunotherapy is the possibility of protecting sensitized individuals from severe allergic response as a consequence of the unintentional exposure to peanuts rather than the reintroduction of peanuts into their diet [78].

Administration sublingually means that droplets or tablets of the allergen are given under the tongue, where they need to stay for a several minutes, and daily allergen doses increase gradually from a submilligram range over a period of days or weeks [81]. In the current study on component analysis in patients undergoing SLIT for peanut allergy, it was confirmed that this diagnostic method allowed them to characterize the molecular sensitization profile, monitor the component-specific effects of peanut immunotherapy, and then make it easier to predict the results of food challenges in patients after 12 months of peanut SLIT [82].

In epicutaneous immunotherapy, allergens are delivered to the skin by skin patches or after abrasion. All patients with a peanut allergy are at risk of an unpredictable life-threatening allergic reaction, but in the youngest children, there is an additional risk factor of accidental exposure to the allergen due to the lack of adequate awareness of the threat. There is no option of treatment for peanut allergy approved for children younger than 4 years. However, the results of a recent multicenter study confirmed the possibility of EIT conducted for 12 months in children 1 to 3 years of age as an effective method in desensitizing children to peanuts, and increasing the dose of peanuts elicited the allergic response [83].

In oral immunotherapy, a dose of peanut allergen in the milligram range is ingested daily and increases over a period of several months. Larger doses of allergens are used in oral immunotherapy compared to sublingual or epicutaneous immunotherapy; therefore, there is a possibility that patients become desensitized to substantial amounts of peanuts. Compared to the effects of inhaled allergen immunotherapy, studies have not confirmed the long-term effectiveness of oral immunotherapy for peanuts [84].

Nowadays, monitoring Ara h 1 and Ara h 2 antibodies may be useful in predicting the effectiveness of peanut oral immunotherapy. Baseline lower concentrations of IgE to Ara h 1–3 are associated with a greater chance of therapy success [85]. People for whom immunotherapy has proven effective had lower IgE levels to peanuts, Ara h 1, and Ara h 2 at baseline and the end of the study compared to those classified as treatment failures [86]. Additionally, lower peanut component-specific IgE to Ara h 6 predicted desensitization [3]. 

According to Tsai et al., patients who originally reacted to a cumulative ingested dose of <500 mg of peanuts and after 117 weeks failed DBPCFCs (double-blind, placebo-controlled, oral food challenges) to 4 g of peanut protein had significantly higher IgEs specific for peanut, Ara h 1, Ara h 2, and Ara h 3 than those who passed the challenge [87]. The repertoire of IgE and IgG4 binding to epitopes of Ara h 1 to 3 undergoes dynamic and personalized changes during OIT and includes a progressive polyclonal increase in IgG4 levels, with the concurrent reduction of IgE amount and diversity. Such changes were not observed in control subjects [88].

The response to OIT may be described as sustained (the amount of peanut tolerated immediately after completing immunotherapy remained the same after a few months of allergen avoidance) or transient (the tolerance decreased).

OIT induces the production of IgG4 subclasses from B cells, and the published literature regarding peanut-specific OIT demonstrated the greatest increase in Ara h 2-specific IgG4 levels compared to the levels of IgG4 directed towards other peanut allergens, indicating the importance of Ara h 2 as a dominant allergen in peanut allergies [89].

Recombinant Ara h 2-specific antibodies cloned from OIT-treated peanut-allergic individuals categorized as either transient or sustained responders were used in a recent study on the mechanism of allergen-specific antibody-mediated tolerance in IgE-mediated peanut allergy.

The authors revealed unique conformational epitopes of Ara h 2, recognized by neutralizing antibodies that effectively impeded allergen-IgE interactions and suppressed basophil degranulation. The induction of these antibodies during OIT may be the explanation of long-lasting allergic tolerance [90]. Moreover, in subjects with sustained unresponsiveness after OIT, a decrease in basophil sensitivity to the immunodominant antigen Ara h 2 was noted, and the basophil area under the curve AUC to Ara h 2 levels after OIT closely correlated with clinical reactivity to peanuts in OIT-treated patients [91].

A recent study conducted on the dose, route, and schedule of administration used methods such as Ara h 1, Ara h 2, Ara h 3, and Ara h 6 quantification to determine the efficacy of prophylactic immunotherapy for peanut allergy and was conducted in an animal model [92]. In this study, rats were administered different doses of peanut protein extract via the oral, sublingual, intragastric, and subcutaneous routes. The results demonstrated that the exposition on peanut allergens via different routes was correlated with different future risks for peanut allergen sensitization and tolerance induction. The results of this type of research conducted on an animal model are currently not sufficient to conclude about the individual risk of peanut allergy or to predict the effectiveness of immunotherapy in individual patients. We still need more studies on peanut immunotherapy as a potential prophylactic strategy to prevent food allergy in population levels. The question of when to start immunotherapy, especially considering AIT in pediatric patients before/at the beginning of atopic march, still remains open. We still need more studies on peanut immunotherapy as a potential prophylactic strategy to prevent food allergy to answer the question of when to start immunotherapy, especially considering AIT in pediatric patients before/at the beginning of atopic march.

Immune tolerance is an active and multifactorial process that may be potentially modulated by various factors. Monoclonal antibodies specific to IgE, such as omalizumab, were explored in combination with oral immunotherapy to increase safety and let the immune system be desensitized more quickly. The results of the Peanut Reactivity Reduced by Oral Tolerance in an anti-IgE Clinical Trial (PRROTECT) revealed the benefits of omalizumab-enabled OIT, such as increasing the safety and tolerability of peanut up-dosing during peanut OIT [93].

There is a need for diagnostic methods that would make it easier to estimate the safety of immunotherapy individually for a given patient, to estimate the significance of additional biological treatment added to immunotherapy, as well as to monitor the success of therapy by biomarkers. CRD may be a useful tool for characterizing the sensitization pattern, including the assessment of the reactivity to peanut allergens, and for choosing the best moment to initiate immunotherapy; therefore, it may be a target for custom-tailored peanut immunotherapy [82].

## 6. Conclusions

Peanut allergy, being one of the most common food allergies, is considered a major health concern worldwide. Its prevalence is increasing, especially in developed countries. Peanut allergy is a serious burden and significantly affects quality of life; thus, prevention, diagnosis, estimation of severity, management, and treatment are crucial. The discovery and description of individual peanut molecules are major milestones that enabled progress in all these areas. In accordance with the conclusions of international allergy societies, CRD can be treated as an equivalent to IgE and SPT in confirming peanut allergy in patients at high clinical risk. Additionally, CRD should be performed in patients with an uncertain peanut allergy history and a positive SPT/sIgE to whole peanuts. CRD is also an appropriate tool for distinguishing between sensitization and co-sensitization to genuine and cross-reactive allergen components. The use of CRD, specifically the measurement of Ara h 6 and Ara h 2 among others, is applied in assessing the risk of a severe allergic reaction. Specific IgE to whole-peanut extract and the magnitude of the SPT do not have such an application. CRD may be useful in predicting peanut immunotherapy effectiveness and durability of tolerance. Assessing unique Ara h 2 epitopes appeared to be helpful in selecting people who can achieve sustained response. Consequently, CRD could help practitioners in identifying good candidates for peanut immunotherapy and those at high risk of adverse reactions.

**Figure 2:**
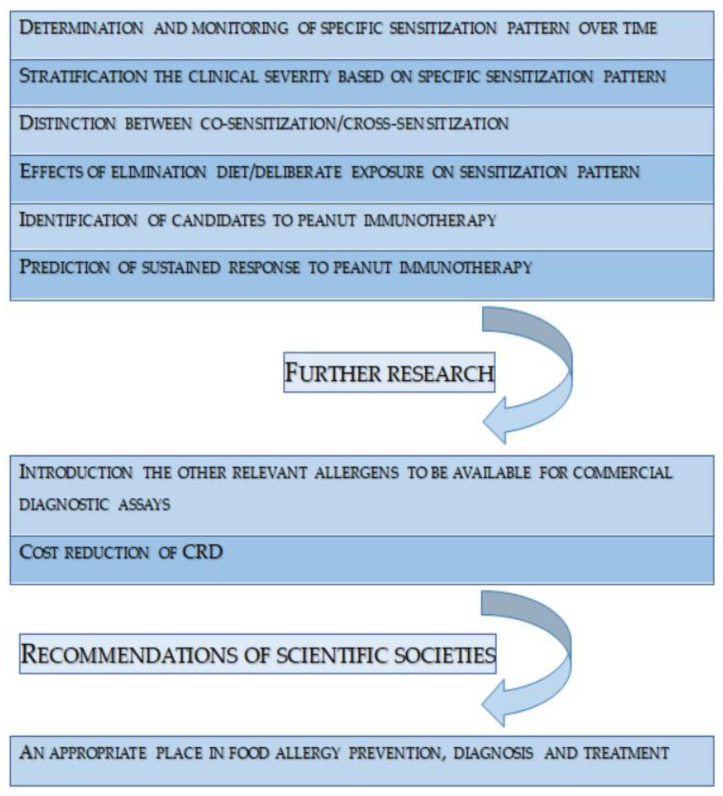
The most relevant concepts in the field of CRD in peanut allergy.

There is still a lack of research on the patient’s profile of sIgE to certain peanut molecules, their sensitivity to specific molecules, and how this profile changes over time depending on exposure to peanuts. It would also be valuable to consider studies based on molecular allergy diagnostic methods that discuss the relationship between a pregnant woman’s exposure to peanuts and the risk of allergy in their child in the future. CRD has a broad application in peanut allergy, in diagnosis, in determining patient management, and in treatment, which is immunotherapy. Further research is certainly necessary to harness the full potential of molecular diagnostics and thus gain additional capabilities in the care of peanut allergy patients (Figure 2).

## Figures and Tables

**Table 1:** Peanut molecules.

Protein Families of Peanuts Molecules	
Ara h 1, Ara h 3	cupins
Ara h 2, Ara h 6, Ara h 7	conglutins
Ara h 5	profilins
Ara h 8	Bet v 1-like
Ara h 9, Ara h 16, Ara h 17	nsLTP
Ara h 10, Ara h 11, Ara h 14, Ara h 15	oleosins
Ara h 12, Ara h 13	defensins
Ara h 18	cyclophilins
